# Adaptation and validation of the university-to-work success scale among Chinese university graduates

**DOI:** 10.3389/fpsyg.2023.1258746

**Published:** 2023-12-06

**Authors:** Qiuping Jin, Kun Yu

**Affiliations:** ^1^School of Labor and Human Resources, Renmin University of China, Beijing, China; ^2^China Institute for Human Capital Audit, Renmin University of China, Beijing, China

**Keywords:** university-to-work success, career adaptability, adult identity, measurement validation, university-to-work transition

## Abstract

**Introduction:**

Whether university graduates successfully make the transition from the university to work is critical for their career development. However, a comprehensive measurement of university-to-work success (UWS) that applies across different contexts remains lacking.

**Methods:**

To address this gap, we adapted and validated the first comprehensive UWS measurement, the university-to-work success scale (UWSS), among samples of Chinese university graduates with three studies. We also provided new construct validity evidence for the scale and examined its measurement invariance across gender.

**Results:**

The findings of the current study showed that the 24-item UWSS-Chinese version clearly showed four factors (career satisfaction, income and financial independence, confidence in career future, and adaptation to work) that were consistent with the original scale. Moreover, construct validity analysis revealed that UWSS was positively associated with proposed antecedents (i.e., career adaptability) and outcome (i.e., adult identity). It also showed incremental validity over general indicators of career success (i.e., career adaptability) in predicting adult identity establishment. Additionally, the measurement also showed measurement invariance across gender.

**Discussion:**

Overall, these findings implied that the UWSS-Chinese version had good psychometric properties to be used in future studies and practice in China.

## 1 Introduction

University-to-work transition is one of the most critical career transitions that university graduates experience in their careers. Whether university graduates successfully make the transition has a significant impact on their career development. Past studies have shown that negative first job experiences increased college students’ risk of long-term decision-making difficulties (Ng and Feldman, 2007), reduced their psychological well-being (Reicherts and Pihet, 2000), and self-efficacy (Fournier and Payne, 1994), and harm their long-term career development and salary prospects (Määttä et al., 2002).

Despite accumulating efforts to understand the contributing factors of university-to-work success (UWS), the model and measurement of UWS have not been well established, impeding progress in this area. The school-to-work-transition (STWT) literature traditionally considers the attainment of employment after graduation as transition success (Koivisto et al., 2007). However, obtaining employment is not enough for the transition to be successful. Once employed, graduates still face multiple challenges to sustain their employment and long-term career development. Based on the role identity theory, graduates need to successfully transit from the student role to the worker role (Ng and Feldman, 2007). From an organizational socialization perspective, fresh graduates also face the transition task of adapting to the organizational culture of their employer (Bauer et al., 2007). Thus, a useful model and measurement of UWS should capture the multiple facets of success that are specific to this stage of career development.

To this end, De Oliveira et al. (2016) proposed a multidimensional model of UWS that encompasses different facets of career success based on the combination of qualitative and quantitative study. They operationalized UWS as the subjective perceptions of intrinsic and extrinsic career outcomes achieved by individuals who have recently completed undergraduate studies. They also developed and validated a measurement, the University to Work Success Scale (UWSS), based on the model. Their model identified four factors that tap different aspects of career success for fresh graduates. The first factor was career insertion and satisfaction, which refers to the obtaining of work in the same field as the degree completed and feelings of career satisfaction. The second factor, “confidence in career future,” refers to feelings of confidence and perseverance required to achieve career goals after graduation. The third factor, “income and financial independence,” taps into graduates’ satisfaction with income and achievement of financial independence from their primary family. Finally, the fourth factor, “adaptation to work,” refers to the adaptation to the demands of the world of work and performance. The model shared some similarities with the traditional general career success model in that they all included perceptions of intrinsic and extrinsic career success outcomes such as career satisfaction, income, and financial rewards. The UWS model, however, identifies two novel intrinsic career success criteria associated with confidence in career future and adaptation to work that were not described before, reflecting the special nature of early career success.

While the model and its measurement are promising in advancing UWS study, they have only been tested in the Brazilian context where they were originally developed. Adapting and validating its measurement for a specific context is important to apply the scale to that context. Adapting and validating the scale in different contexts is also crucial to establish the contextual generalizability of the scale. To this end, our current study aims to adapt and validate the scale for use with Chinese university graduates, a group that consists of more than 10 million individuals each year and who has consistently shown difficulties in this critical career transition (Knight et al., 2017; Hao and Wang, 2022). Moreover, in terms of the psychometric property of the scale, the original scale development study only provided the convergent validity of the scale with salary. It’s important to provide more psychometric evidence in supporting the scale for further application of the scale in future studies. In light of this, the second aim of our study is to examine the measurement invariance of the scale for gender and test its construct validity about its theoretically proposed antecedents and outcomes.

## 2 Literature review and research questions

### 2.1 University-to-work transition and success in the Chinese context

Since higher education expansion in 1999, China’s higher education system has rapidly evolved from elite education to mass education (Mok and Wu, 2016). In 2023, China is expected to have 11.58 million new college graduates, up by 820,000 from last year (Ministry of Education of China, 2023). However, the university-work transition for Chinese students has proven to be challenging due to multiple intricate reasons. For one thing, China’s education is typically seen as examination-oriented (Dello-Iacovo, 2009), emphasizing academic achievement instead of exploring the self and personal interests. Chinese university students have reported low levels of career exploration and career goal setting compared to their counterparts in the West (Zhang et al., 2018), contributing to the difficulty of adapting to the world of work. A survey showed that around one-third of Chinese university graduates from 2017 to 2019 left their first job within half a year (MyCos Research Institute, 2019) due to difficulties adapting to their jobs and low job satisfaction. Studies of UWS in China are thus of significant practical importance. However, there were only very limited studies on UWS in China. The few existing studies adopted diverse conceptualizations for UWS, including achieving employment and employment satisfaction (Zhang and Dong, 2011) and person-job fit (Tan and Qiu, 2017). Adapting the UWSS to provide a comprehensive and valid measurement for UWS in China can contribute to stimulating more studies in the area.

UWS was defined as the subjective perceptions of intrinsic and extrinsic career outcomes achieved by individuals who have recently completed undergraduate studies. The four-factor UWS model by De Oliveira et al. (2016) captured all the essential components of success during the transition from school to work, thus it shall also apply across contexts. Similar to the general model of career success, the model included the perception of intrinsic career success, that is, career satisfaction. It also included perceptions of the extrinsic success of income and financial rewards, emphasizing income and financial independence from the original family to better reflect the financial success definition of this stage of life. In addition, it also has a factor of adaptation to work that captures the success in the task of transition from the student role to the worker role, a task that is characteristic of school-to-work transition (Ng and Feldman, 2007). Finally, fresh graduates are at the beginning stage of their career establishment, a successful beginning should give them confidence in their future while failure of such may diminish their perception of their career outlook (Koen et al., 2012). Thus, a successful transition should give them confidence in the future, a factor also included in the UWS model.

Despite the comprehensiveness and contextual generalizability of the model, there are also potential contextual differences that may also cause the UWS experience to be unique in the Chinese context and that require revisions of the original UWSS items to reflect such differences. First, the factor of “career insertion and satisfaction” of the UWSS views “obtaining work in the same field as one’s degree” as an indication of transition success. In contexts where university students choose the field of study according to their career interests and where they can change their field of study with relative ease, working in the degree field may indeed capture outcomes resulting from individuals’ efforts in the pursuit of one’s career goal. In the Chinese context, however, university students choose a college major for diverse reasons other than one’s interest. A study reported that Chinese students choose a major to fulfill their parental requirements as much as to pursue their career interests (Yu, Yu et al., 2018). Changing academic majors during university also faces many restrictions since the number of students studying in each major in a particular university is predetermined by the government. As a result, major-job mismatch is not uncommon. National scale surveys of university graduates in recent years have consistently shown that around a third of Chinese university graduates work in a job that does not relate to their field of study (Yue, 2017). More importantly, a study also showed that unlike in other contexts, major-job mismatch did not necessarily lead to income loss for university graduates in China (Zhu, 2014). For a proportion of individuals, it even led to an income increase. Due to these reasons, we argue that career insertion may not be a good reflection of UWSS in the Chinese context, and items measuring career insertion are not to be included in the Chinese version of UWSS.

Further, some items of the other factors may not reflect the reality in China either. One item of the “income and financial independence” factor was “I am able to live by myself (out of my parents’ house).” In the Chinese context, because of its vast geographic distribution, mobility is very high since its economic reform. A large proportion of young people in China leave their hometowns for big cities and better-developed areas to seek study and work opportunities (Feng et al., 2017). Thus, living by oneself outside of parents’ house is usually not out for reasons of gaining independence but merely an arrangement forced by reality. Based on this, it would provide a poor indication of the factor and we also take out this item from the original scale to adapt the scale for the Chinese context.

### 2.2 Validity evidence of the UWSS

The original study only provided the convergent validity of the UWSS with salary. To validate the scale, further evidence was needed. Following the widely accepted criteria suggested by the Standards for Educational and Psychological Testing (AERA et al., 2014), various sources of validity evidence can be used to evaluate the validity of an adapted scale. In the present study, we aimed to obtain three sources of evidence: evidence based on content validity, evidence based on internal structure, evidence based on relations with other variables, and evidence of the measurement invariance across genders. With revisions and adaptions of the scale for the Chinese context, the content validity of the revised scale will need to be examined. Internal structure refers to the dimensionality or the factor structure underlying the UWSS. For the revised scale, we will examine the factor structure of the scale with a combination of exploratory factor analysis (EFA) and confirmatory factor analysis (CFA).

Construct validity evidence concerning the UWS score’s relations with theoretically proposed antecedents and outcomes is lacking. Previous studies have identified that career adaptability is one of the most important resources for individuals to cope with the task of transitioning into the world of work (e.g., De Oliveira et al., 2016). Career adaptability, by its definition, refers to individuals’ psychosocial resources to cope with anticipated and real career transitions (Savickas, 2005). Career adaptability has been shown to predict various indicators of the university to work transition success, including job search self-efficacy, employment status (Guan et al., 2014), employment quality (Koen et al., 2012), and life satisfaction (Hlaďo et al., 2022). Career adaptability score should therefore be positively related to UWSS score.

To be a valid measurement, the UWSS score should also be able to predict outcomes closely related to school-to-work transition success. Based on developmental psychology theories, adult role transitions including completion of education, entrance to the job market, and attainment of financial independence are makers of adult identity achievement (Benson and Elder, 2011). Empirical research did confirm that adult role transitions are reliable predictors of adult identity establishment (Eliason et al., 2015; Piotrowski et al., 2020). Specifically, completing formal education and getting a job were found to facilitate adult identity commitment (Luyckx et al., 2008), while experiencing difficulties in the job market were more often related to problems with identity commitment formation (De Goede et al., 1999; Danielsen et al., 2000).

UWS reflects these adult role transitions and should predict adult identity. Developmental and life course studies of young adult identities identified two dimensions of subjective perception of an adult identity, subjective age and psychosocial maturity (Arnett, 2000; Shanahan et al., 2005). Subjective age is based on social comparisons and self-perceptions, such as how old youth perceive themselves to be in comparison with peers of the same chronological age and whether they identify with a certain age group (Shanahan et al., 2005; Johnson et al., 2007). Psychosocial maturity focuses on intra-individual development and examines the psychosocial maturation aspects of identity, such as independence, confidence, and responsibility (Arnett, 2000). It can be seen from the definitions that the dimensions of confidence in the future and income and financial independence of the UWS are also the key features of psychosocial maturity and successful adapting to the worker role should signify older subjective age compared to those who experience poor adaptation. Further, as discussed earlier, developmental psychology theories hold that adult role transitions including obtaining employment are important makers of adult identity achievement (Benson and Elder, 2011). The contribution of career adaptability to employment obtainment has been well documented in the literature (e.g., Koen et al., 2012; Guan et al., 2014), and employment obtainment, in turn, predicts adult identity. Thus, career adaptability should also contribute to adult identity achievement. However, UWS captures aspects of career success that are specific to the stage of transition to adulthood, including confidence in the future and income and financial independence while career adaptability predicts career success more generally. Thus, the impact of UWS on adult identity should exist even after controlling for general career resources and success indicators such as career adaptability, suggesting incremental validity of UWSS over general career success indicators. Overall, we propose to evaluate the construct validity by testing the relations of UWSS scores with career adaptability and adult identity.

The evaluation of the measurement invariance of a scale across groups is also important because it determines whether each item used in the instrument means the same thing to participants of different groups (Cheung and Rensvold, 2002). If there is no measurement invariance across groups, then among groups differences in scores cannot be unambiguously interpreted (Cheung and Rensvold, 2002). In terms of group differences in career success, previous studies have paid special attention to gender differences. Based on gender role theory, traditional female roles are considered to attach more importance to family caring while male roles attach more importance to achievement when setting their career goals (Stuhlmacher and Poitras, 2010). Thus, traditionally minded females and males may differ in how they perceive career success in general and UWS specifically. Due to economic modernization and policies to promote gender equality, gender differences may have diminished in China (Skromme Granrose, 2007). Empirical studies, however, led to inconsistent findings about whether gender differences in career goals and values still prevail in China (Jiang and Yang, 2011; Yi et al., 2015). Thus, it is necessary to assess if and to what extent the UWSS items mean the same thing to both genders in the Chinese context by testing the measurement invariance of scale, including configural, metric, and scalar invariance.

### 2.3 The current study

To summarize, the current study aims to adapt and validate the UWSS scale for use in the Chinese context. We achieved our aims in three studies. We first conducted a pilot study to examine the content validity of the translated and adapted UWSS scale with experts in the area and students experiencing university-to-work transition. In the second study, we explored the factor structure of the revised with EFA. In the third study, we examined the factor structure of the revised scale from Study 1 and its measurement invariance for gender with CFA. To provide further construct validity evidence of the scale, we also tested the relations of the scale’s score with career adaptability and adult identity.

## 3 Study 1 scale adaption

In this study, we aimed to adapt the UWSS and examined the content validity of the adapted scale. The UWSS has 22 items and is composed of four dimensions. Based on the above discussions of the contextual understanding of university-to-work transition in China, three items that we argued were inappropriate to reflect UWS in China were deleted from the scale and were not included in the study. Among the three, two items measured the fit between graduates’ field of degree and the work, namely “I am working in the same field of my degree” and “I am involved somehow with my degree area (e.g., employment, job enhancement, post-graduate studies).” The other one aimed to measure income and financial independence but may fail to capture this characteristic in China, namely “I am able to live by myself (out of my parents’ house).” Furthermore, to avoid having too few items after potential item deletion in the next step of content validity examination and EFA, we added in the items that De Oliveira et al. (2016) produced in their item generation study but were not included in their final 22 item scale. The original item pool of Oliveira’s study was derived in an inductive qualitative manner based on the definition of UWS and was agreed upon by experts in the field. A close examination of the deleted items showed that they were deleted in the EFA step in a data-driven manner. It was likely that these items were valid in capturing UWS factors but were deleted due to language or chance factors of the data. Thus, it was appropriate to add these items for further exploration in this adaptation, especially considering this will prevent having too few items after further potential deletion of items. Moreover, items added that might be inappropriate would be screened out in the content validation examination step. The addition of these items resulted in 27 items. The 27-item scale was then translated from English to Chinese following the Translation-back-translation procedure recommended by Brislin (1980). Specifically, the 27 items were first translated into Chinese by a specialist in the area. Then all Chinese items were back-translated into English by another specialist. Finally, the original items and the items back-translated into English were matched by a third specialist. The scale was rated from 1 (Strongly disagree) to 7 (Strongly agree) with higher scores representing higher levels of the university to work success.

After scale translation, the second task was to demonstrate evidence for content validity. To achieve this goal, the list of 27 items generated in the previous step was sent to be reviewed by two experts, psychology PhDs who were experts in the study of university-to-work transition, career success, and measurement construction. The judges were instructed to evaluate each item regarding language clarity, and practical and theoretical relevance. In addition, the reviewers were asked to contribute suggestions about the items and their contents. The two experts judged the items to be appropriate to reflect UWS in China and did not suggest further revisions to the scale. The scale was then provided to a group of 10 recent university graduates to assess the clarity and relevance of the items and scale instructions. The participants were approached through snowball sampling. They were also purposefully chosen to be balanced as much as possible in terms of gender, major of study, and sector of employment. Their participation was voluntary and were given no monetary rewards. They were made aware of the purpose of the scale and filled out the scale. Afterward, each of them discussed the clarity and relevance of the items with the two authors together. All the participants indicated that the language and instruction of the scale were easily understood by them and all the items were relevant to their school-to-work transition experiences. They suggested no revisions of the scale. Thus, the results of both expert and target group studies provided evidence for the items’ content validity.

## 4 Study 2 exploratory factor analysis

### 4.1 Method

#### 4.1.1 Participants and procedures

Data in this study were collected via Credamo, a platform in China that provides functions equivalent to Amazon Mechanical Turk. Studies that collected data from the platform were published in peer-reviewed journals such as the Journal of Business Ethics (Zhou et al., 2022) and Personality and Individual Differences (Chen et al., 2022). Surveys were distributed to a total of 400 employees who have graduated from universities for a period of 6 months to 42 months. The time period was selected following previous studies of university-to-work transition (e.g., Bauer et al., 2007; De Oliveira et al., 2016). 370 completed questionnaires were received (a response rate of 92.5%). Participants’ age on average was 23.08 years (*SD* = 1.72), and average tenure was 18.37 months (*SD* = 9.13). Among all 370 participants, 136 (36.8%) were male, and 234 were female (63.2%).

#### 4.1.2 Measures

University to work success-Chinese version. University-to-work success was assessed with the 27-item Chinese scale adapted and evaluated in the pilot study. These items were rated from 1 (Strongly disagree) to 7 (Strongly agree) with higher scores representing higher levels of the university to work success. Sample items were “I am working in the same field of my degree “, “I am financially independent from my family,” “I am confident about my professional future” and “I get compliments on my professional performance.”

#### 4.1.3 Analysis

To provide statistical characteristics of the items and test the content validity of the scale (Merino-Soto et al., 2022), we performed item analyses using descriptive statistics such as response frequency, and response similarity. Moreover, to assess the latent factor structure of the UWSS measure, we utilized Exploratory factor analysis (EFA) and calculated the factor loadings of scale items (Tabachnick and Fidell, 2007).

#### 4.1.4 Results

We first performed an item analysis. As can be seen in Table 1, the results of response frequency indicated that option 5 (somewhat agree) has the highest response frequency across 25 items, except item 6 (with 6 (agree) has the highest response frequency) and item 13 (with 4 (either agree or disagree) has highest response frequency), while option 1 (strongly disagree) showed lowest prevalence in most items. Moreover, the summary descriptive statistics indicate a mean of 4.92 and a high mean response similarity (MaxM/MinM = 1.38). The distributions were all negatively skewed.

**Table 1:** Item analyses of Study 2.

Response frequency of UWSS items								Descriptive statistics				
Item	1 (Strongly disagree)	2 (Disagree)	3 (Somewhat disagree)	4 (Either agree or disagree)	5 (somewhat agree)	6 (Agree)	7 (Strongly agree)	Mean	SD	Mode	Skewness	Kurtosis
UWS1	4	10	28	56	109	98	65	5.19	1.344	5	−0.638	0.15
UWS2	6	19	37	60	122	75	51	4.9	1.426	5	−0.506	−0.125
UWS3	1	4	16	64	134	108	43	5.22	1.094	5	−0.436	0.339
UWS4	12	12	45	65	102	87	47	4.84	1.482	5	−0.561	−0.1
UWS5	6	18	40	53	104	89	60	4.99	1.472	5	−0.564	−0.258
UWS6	4	7	23	38	93	108	97	5.49	1.342	6	−0.904	0.586
UWS7	9	8	37	63	117	90	46	4.96	1.378	5	−0.633	0.284
UWS8	8	10	27	54	108	106	57	5.14	1.384	5	−0.799	0.503
UWS9	8	6	23	44	112	112	65	5.28	1.339	5	−0.936	1.003
UWS10	11	7	23	51	107	104	67	5.21	1.415	5	−0.911	0.806
UWS11	20	19	29	73	107	79	43	4.72	1.564	5	−0.651	−0.019
UWS12	13	23	51	86	103	63	31	4.5	1.473	5	−0.338	−0.301
UWS13	33	32	68	91	87	37	22	3.99	1.584	4	−0.143	−0.529
UWS14	10	13	22	59	115	109	42	5.03	1.378	5	−0.878	0.72
UWS15	7	11	37	71	122	77	45	4.89	1.36	5	−0.496	0.115
UWS16	2	6	10	54	135	104	59	5.33	1.14	5	−0.617	0.862
UWS17	6	4	23	44	119	112	62	5.3	1.27	5	−0.868	1.033
UWS18	7	16	29	55	98	94	71	5.13	1.472	5	−0.693	0.013
UWS19	15	21	35	77	97	82	43	4.72	1.537	5	−0.547	−0.191
UWS20	16	21	39	59	115	77	43	4.73	1.546	5	−0.599	−0.146
UWS21	10	18	37	64	102	96	43	4.86	1.468	5	−0.622	−0.071
UWS22	21	27	64	79	93	60	26	4.3	1.558	5	−0.274	−0.53
UWS23	17	28	39	69	99	84	34	4.6	1.571	5	−0.535	−0.374
UWS24	24	29	56	68	102	65	26	4.34	1.598	5	−0.383	−0.558
UWS25	5	5	17	45	128	124	46	5.28	1.185	5	−0.951	1.539
UWS26	6	8	38	78	125	85	30	4.85	1.269	5	−0.508	0.285
UWS27	9	8	24	55	127	100	47	5.08	1.33	5	−0.843	0.905

**Figure 1:**
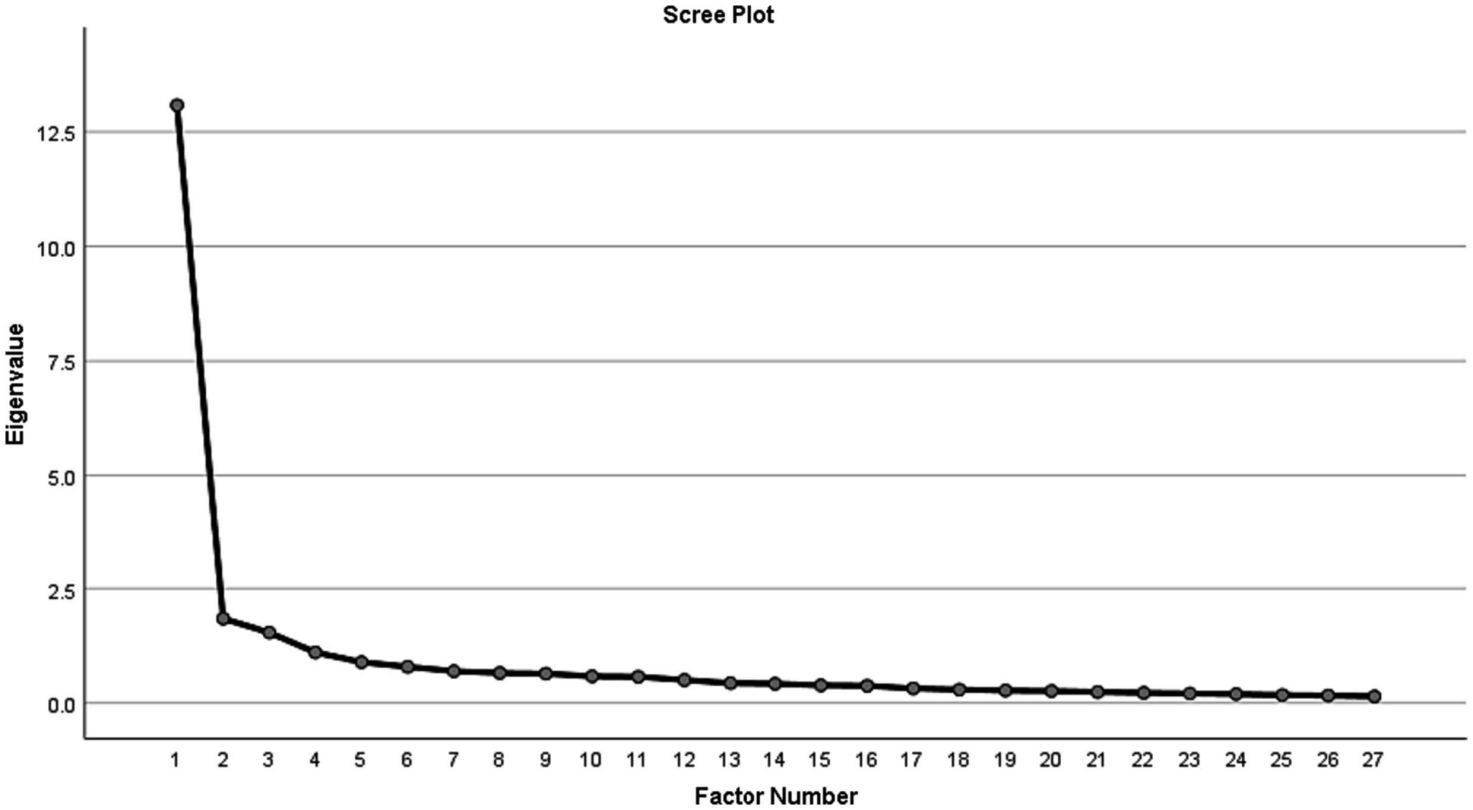
Scree plot analysis of Study 2.

Next, EFA using the maximum likelihood (ML) extraction method with Oblimin rotation was performed in Mplus 8. The Kaiser-Meyer-Olkin measure of sample adequacy was 0.954, which was higher than the recommended 0.60 cutoff value (Worthington and Whittaker, 2006), and Bartlett’s test of sphericity was significant (*p* < 0.001), indicating that the UWSS scale was suitable for factor analysis. The number of factors to be retained is determined by inspecting the scree plot, discontinuities between factors, the number of eigenvalues greater than 1, and the interpretability of the factor solution. As can be seen in Figure 1, the results revealed that four factors have eigenvalues greater than 1. We then examined rotated solutions of four-, three-, two- and one-factor structures. Thurstone’s (1947) criterion of simple structure was used to determine the most plausible factor structure. According to Thurstone (1947), to be a simple structure, that is easily interpretable, meaningful, and replicable, there should be high within-factor loading variability and low factorial complexity in defining variables. The three-, two- and one-factor solutions failed to meet the criterion of simple structure. Specifically, the three-factor structure had a weak factor composed of only 5 items, compared to another factor of 12. For the two-factor model, there was also a weak factor, with strong loadings to only 5 items, compared to the other factor of 21. The one-factor model only accounted for 48.44% of the estimated common variance, below the 60% cut-off value recommended by Hair et al. (2009). Compared to other structures, the four-factor solution accounting for 65.04% of the estimated common variance provided the most meaningful result, as presented in Table 2.

**Table 2:** Exploratory factor analyses of the university-to-work success scale (27 items) in Study 2.

Items	Factor loading			
	1	2	3	4
1 I can pay my bills	−0.114^*^	0.862^*^	0.008	0.011
2 I am able to buy things that interest me	0.171^*^	0.754^*^	−0.046	−0.069
3 I am meeting the requirements of the world of work	−0.042	0.268^*^	0.516^*^	0.061
4 I am working on what I have planned	0.314^*^	0.131	0.068	0.312^*^
5 I am confident about my professional future	0.251	0.145	−0.035	0.492^*^
6 I am financially independent from my family	0.049	0.519^*^	0.067	0.066
7 I get remuneration compatible with the labor market in my area	−0.035	0.610^*^	0.102	0.159
8 I have good expectations about my career future	0.089	0.157	−0.047	0.756^*^
9 I have had patience to achieve my career goals	0.032	−0.029	0.117	0.784^*^
10 I feel reassured about my career future	0.013	0.011	0.043	0.823^*^
11 I was promoted (or rewarded) due to my job performance	0.378^*^	0.166^*^	0.333^*^	−0.079
12 I get a good pay compared to professionals in my field	0.258^*^	0.515^*^	0.105	0.03
13 I feel like a professional in my field	0.327^*^	0.001	0.421^*^	0.032
14 I am confident that I will be able to achieve my career goals	0.074	−0.057	0.234^*^	0.671^*^
15 I am adapted to the culture of the workplace (organizational culture)	0.158	0.11	0.487^*^	0.044
16 I have been achieving the satisfaction of people who need or make use of the results of my work	−0.118^*^	0.082	0.679^*^	0.161
17 I get compliments on my professional performance	0.144	−0.065	0.637^*^	0.102
18 I have been referred by people I know to work opportunities	0.017	−0.122	0.511^*^	−0.051
19 I am working on what I like	0.658^*^	−0.016	−0.044	0.261
20 I am satisfied with my career after graduation	0.681^*^	0.065	−0.047	0.257
21 I am satisfied with my job	0.676^*^	0.07	0.039	0.183
22 I feel accomplished in my profession	0.764^*^	0.022	0.198^*^	−0.125^*^
23 I am happy with my achievements after graduation	0.837^*^	0.029	0.037	−0.021
24 I have succeeded in achieving the goals I have planned for my career	0.835^*^	0.018	−0.006	0.04
25 I am adapted to the requirements and responsibilities of my job	0.147	0.151	0.423^*^	0.108
26 I get into the world of work	0.158	0.191^*^	0.406^*^	0.069
27 I have been socially recognized by my professional performance	0.169^*^	0.024	0.505^*^	0.212^*^
Eigenvalues	13.079	1.842	1.537	1.103
% Explained variance (cumulative)	48.44	55.26	60.95	65.04

*N* = 370. Extraction Method: Principal Axis Factoring method with rotation using Oblimin with Kaiser Normalization.

* Indicates *p* < 0.05.

According to Beavers et al. (2013) and Hinkin (1998), items with cross-loadings or loadings less than 0.40 should be deleted. First, item 11 (“I was promoted (or rewarded) due to my job performance”) had double loadings on factor 1 (factor loading = 0.357) and factor 3 (factor loading = 0.303). The first factor reflected “career satisfaction” and the third factor reflected “adaptation to work.” Indeed, getting promoted can be a source of career satisfaction as well as an indication that one has adapted to the workplace which enabled good performance. Item 13 (“I feel like a professional in my field”) also had double loading on factor 1(factor loading = 0.308) and factor 2 (factor loading = 0.408). The second factor reflected “income and financial independence.” “Feeling like a professional” may have different connotations for individuals with different career values. For those who value personal development, such a feeling may contribute to their sense of career satisfaction while for those who value momentary rewards, it may imply to them that they are doing well financially. For such reasons, both item 11 and 13 were deleted from the scale. Moreover, item 4 (“I am working on what I have planned”) had no loading higher than 0.4 on any factor. Grounded on Beavers et al. (2013), this item was deleted to keep a measure short and minimize response biases caused by boredom or fatigue. To sum up, items 4, 11, and 13 were deleted from the scale, and 24 items remained.

Next, EFA using the maximum likelihood (ML) extraction method with Oblimin rotation was again performed in Mplus 8 on the remaining 24 items. Table 3 illustrates the final factor loadings for each item, clustered on 4 factors. All the factor loadings were above 0.4. The four-factor structure accounted for 67.07% of the total variance and provided a parsimonious and simple factor structure that could be readily interpreted. Consistent with De Oliveira et al. (2016), the first factor was named “career insertion and satisfaction” (CIS)and consisted of 6 items, the second factor was named “income and financial independence” (IFI) and contained 5 items, and the third factor was named “adaptation to work” (AW)and consisted of 8 items, and the fourth factor was named “confidence in career future” (CCF), which consisted of 5 items. All four factors of the 24-item scale had good internal consistency coefficients, for CIS, Cronbach’s α =0.94, McDonald’s omega = 94; for IFI, Cronbach’s α =0.85, McDonald’s omega = 0.85; for WA, Cronbach’s α = 0.86, McDonald’s omega = 0.86; and for CCF, Cronbach’s α = 0.92, McDonald’s omega = 0.92. Moreover, As shown in Table 4, the interfactor correlations for the four factors were strong and statistically significant.

**Table 3:** Results of exploratory factor analyses of the university-to-work success scale (24 items) in Study 2.

Items	Factor loadings			
	1	2	3	4
Factor 1: career insertion and satisfaction (CIS)				
19 I am working on what I like	0.685^*^			
20 I am satisfied with my career after graduation	0.713^*^			
21 I am satisfied with my job	0.714^*^			
22 I feel accomplished in my profession	0.766^*^			
23 I am happy with my achievements after graduation	0.852^*^			
24 I have succeeded in achieving the goals I have planned for my career	0.849^*^			
Factor 2: income and financial independence (IFI)				
1 I can pay my bills		0.853^*^		
2 I am able to buy things that interest me		0.756^*^		
6 I am financially independent from my family		0.502^*^		
7 I get remuneration compatible with the labor market in my area		0.591^*^		
12 I get a good pay compared to professionals in my field		0.505^*^		
Factor 3: adaptation to work (AW)				
3 I am meeting the requirements of the world of work			0.543^*^	
15 I am adapted to the culture of the workplace (organizational culture)			0.499^*^	
16 I have been achieving the satisfaction of people who need or make use of the results of my work			0.708^*^	
17 I get compliments on my professional performance			0.646^*^	
18 I have been referred by people I know to work opportunities			0.482^*^	
25 I am adapted to the requirements and responsibilities of my job			0.490^*^	
26 I get into the world of work			0.439^*^	
27 I have been socially recognized by my professional performance			0.483^*^	
Factor 4: confidence in career future (CCF)				
5 I am confident about my professional future				0.499^*^
8 I have good expectations about my career future				0.771^*^
9 I have had patience to achieve my career goals				0.798^*^
10 I feel reassured about my career future				0.861^*^
14 I am confident that I will be able to achieve my career goals				0.697^*^
Eigenvalues	11.748	1.821	1.511	1.017
% Explained variance (cumulative)	48.95	56.53	62.83	67.07

*N* = 370. Extraction Method: Principal Axis Factoring method with rotation using Oblimin with Kaiser Normalization.

* Indicates *p* < 0.05.

**Table 4:** Results of interfactor correlations in Study 2.

	Factor 1	Factor 2	Factor 3	Factor 4
Factor 1	-			
Factor 2	0.49^***^	-		
Factor 3	0.57^***^	0.40^***^	-	
Factor 4	0.73^***^	0.43^***^	0.63^***^	-

*N* = 370. ***Indicates *p* < 0.001.

## 5 Study 3 confirmatory factor analysis and construct validity

### 5.1 Method

#### 5.1.1 Participants and procedures

Data from this study were also collected via Credamo. Surveys were distributed to a total of 400 employees who graduated from universities from 6 months to 42 months. Finally, 384 completed questionnaires were received (a response rate of 96%). Participants’ age on average was 23.52 years (SD = 1.54), and average tenure was 19.01 months (*SD* = 9.47). Among all 384 participants, 140 (36.5%) were male, and 244 were female (63.5%).

#### 5.1.2 Measures

*University-to-work success* was assessed using the 24-item University-to-work success scale (UWSS) adapted in Study 2. These items were rated from 1 (*Strongly disagree*) to 7 (*Strongly agree*) with higher scores representing higher levels of the university to work success. The alpha coefficient of the current scale was 0.91 for CIS, 0.81 for IFI, 0.83 for WA, 0.91 for CCF, and 0.95 for the whole scale.

*Career adaptability* was assessed using Maggiori et al. (2015)’s Career Adapt-Abilities Scale-Short Form (CAAS-SF). The validity of the CAAS-SF was supported by recent studies (e.g., Yu et al., 2020). The 12-item scale is composed of four dimensions (concern, control, curiosity, and confidence). Participants were asked to evaluate the strength of their abilities. Sample items were “Preparing for the future,” “Making decisions by myself,” “Looking for opportunities to grow as a person,” and “Taking care to do things well” for the four dimensions, respectively. These items were rated from 1 (*very weak*) to 7 (*very strong*), with a higher score representing a higher level of career adaptability. The alpha coefficient of the career adaptability scale was 0.87.

*Adult identity* was assessed using the four-item scale developed by Benson and Elder (2011), containing two dimensions of subjective age and psychosocial maturity. Participants were asked to rate their subjective age, rate of growth, level of social maturity, adult responsibilities, and adult status. Items were “How old do you feel compared with others your age?” “How fast they grow up regarding their same-aged peers?” “How often do you think of yourself as an adult?” and “How do you think the word independence, confidence, and considerateness are suitable to describe you?” Among the four items, the first was rated from 1 (younger all of the time) to 5 (older all of the time), the second item (reverse coded) was rated from 1 (much faster) to 5 (much slower), the third was rated from 1(never) to 5 (all of the time), and the last was rated from 1(not at all) to 5 (very). The adoption of a subjective measurement of adulthood in the current study has the advantage over the objective measurement of attaining adulthood status such as being married in that nowadays young people adopt very different objective markers of adult status (Bao et al., 2023). Though the current scale has not been used in the Chinese population in its entirety, the item of the perception of adult status has been applied in China and has shown good reliability and validity (Bao et al., 2023). Moreover, a study comparing Chinese and US adults found that with globalization young people of the Chinese culture attached a similar meaning of adulthood to the US youths, giving further confidence that the scale applied to the Chinese culture too (Nelson et al., 2013). The alpha coefficient of the current scale was 0.63.

### 5.2 Results

#### 5.2.1 Factor structure

To further assess the factor structure of the UWSS in Chinese employees, we performed an Explore Structural factor analysis (ESEM) using Mplus 8 (Muthén and Muthén, 1998–2017). Three models were examined and compared, including four-, three-, two, and single-factor models. The results of the model fit of the four models are summarized in Table 5. The four-factor model, with *χ*^2^(186) = 381.415, *p* < 0.01, CFI = 0.951, TLI = 0.927, RMSEA = 0.052, SRMR = 0.027, AIC = 25619.002, and 26164.190, fitted the data better than the three-factor model [*χ*^2^(207) = 559.198, *p* < 0.01, CFI = 0.912, TLI = 0.883, RMSEA = 0.067, SRMR = 0.039, AIC = 25784.006, and BIC = 26246.231], the two-factor model [*χ*^2^(229) = 751.634, *p* < 0.01, CFI = 0.869, TLI = 0.843, RMSEA = 0.077, SRMR = 0.045, AIC = 26008.821, and BIC = 26384.132], and the single-factor model [*χ*^2^(252) = 986.687, *p* < 0.01, CFI = 0.816, TLI = 0.799, RMSEA = 0.087, SRMR = 0.064, AIC = 26341.108, and BIC = 26625.554] across all indices. Moreover, As shown in Table 6, the interfactor correlations for the four factors were strong and statistically significant.

**Table 5:** Results of the confirmatory factor analysis in Study 3.

MODEL	*χ^2^*	*df*	*χ^2^/df*	CFI	TLI	RMSEA	SRMR	AIC	BIC
Four-factor	381.415	186	2.051	0.951	0.927	0.052	0.027	25619.002	26164.190
Three-factor	559.198	207	2.701	0.912	0.883	0.067	0.039	25784.006	26246.231
Two-factor	751.634	229	3.282	0.869	0.843	0.077	0.045	26008.821	26384.132
Single-factor	986.687	252	3.915	0.816	0.799	0.087	0.064	26341.108	26625.554

*N* = 384.

**Table 6:** Results of interfactor correlations of UWSS in Study 3.

	Factor 1	Factor 2	Factor 3	Factor 4
Factor 1	-			
Factor 2	0.75^***^	-		
Factor 3	0.81^***^	0.69^***^	-	
Factor 4	0.81^***^	0.70^***^	0.75^***^	-

*N* = 384. ***Indicates *p* < 0.001.

#### 5.2.2 Measurement invariance tests

To further test the measurement invariance of UWSS across genders, we conducted a series of multiple-group confirmatory factor analyses (CFA), including configural, metric, and scalar invariance as recommended by Vandenberg and Lance (2000), using Mplus 8 (Muthén and Muthén, 1998–2017). For gender (male and female), results of configural invariance indicated a satisfactory model fit, *χ*^2^(492) = 1016.895, *p* < 0.01; CFI = 0.91, RMSEA = 0.075, which suggested the factors and pattern structure of UWSS scale were equivalent across gender. We then tested model comparisons with more restrictive levels of invariance imposed in each step across gender. To test metric invariance, we compared the fit of the metric invariance model which constrains all factor loadings to be equal to the fit of the configural model. Results revealed that *χ*^2^difference statistic [Δ*χ*^2^(20) = 28.131, *p* > 0.05] was non-significant, which assumed an equal factor loading across gender. Next, to test scalar invariance, we compared the fit of the scalar invariance model which requires item intercepts to be equivalent to the fit of the metric model. We found that, although χ2 difference statistic [Δ*χ*^2^(31) = 84.012; *p* < 0.001] was significant, we also found that ΔCFI = 0.009, and ΔRMSEA = 0.001. As scholars recommended that a ΔCFI smaller than or equal to 0.01 and a ΔRMSEA up to 0.01 or 0.015 indicate invariance (Cheung and Rensvold, 2002; Chen, 2007), the results demonstrated that item intercepts were invariant across gender.

#### 5.2.3 Construct validity estimates

To provide evidence of UWSS’s validity, as discussed above, we examined if UWSS scores were related to its theoretically proposed antecedents (i.e., career adaptability) and outcome (i.e., adult identity) by performing a set of Structural Equation Modeling (SEM) using Mplus 8 (Muthén and Muthén, 1998–2017). First, means, standard deviations, and correlations between research variables can be seen in Table 7. Next, results of confirmatory factor analysis (CFA) revealed that the three-factor model (UWSS, adult identity and career adaptability independent from each other) had better model fit [*χ*^2^(704) = 1288.247, *p* < 0.01; CFI = 0.91, TLI = 0.90, RMSEA = 0.046, SRMR = 0.049] than the single-factor model [*χ*^2^(740) = 2263.299, *p* < 0.01; CFI = 0.76, TLI = 0.75, RMSEA = 0.065]. Next, to test the measurement invariance of UWSS and related constructs across gender, we conducted a series of multiple-group confirmatory factor analyses (CFA), including configural, metric, and scalar invariance as recommended by Vandenberg and Lance (2000), using Mplus 8 (Muthén and Muthén, 1998–2017). For gender (male and female), results of configural invariance indicated a satisfactory model fit, *χ*^2^(1408) = 2374.739, *p* < 0.01; CFI = 0.87, RMSEA = 0.060, which suggested the factors structure of UWSS and related constructs were equivalent across gender. We then tested model comparisons with more restrictive levels of invariance imposed in each step across gender. To test metric invariance, we compared the fit of the metric invariance model which constrains all factor loadings to be equal to the fit of the configural model. Results revealed that χ^2^ difference statistic [Δ*χ*^2^(21) = 16.484, *p* > 0.05] was non-significant, which assumed an equal factor loading across genders. Next, to test scalar invariance, we compared the fit of the scalar invariance model which requires item intercepts to be equivalent to the fit of the metric model. We found that, although χ^2^ difference statistic [Δ*χ*^2^(57) = 177.574; *p* < 0.001] was significant, we also found that ΔCFI = 0.017, and ΔRMSEA = 0.002. As scholars recommended that a ΔCFI smaller than or equal to 0.01 and a ΔRMSEA up to 0.01 or 0.015 indicate invariance (Cheung and Rensvold, 2002; Chen, 2007), the results demonstrated a considerable level of scalar invariance. Furthermore, to test the relations of UWS with its antecedents, we performed a set of SEM analyses with career adaptability as the independent variable, and UWS as the dependent variable. As can be seen in Table 8, the effect of career adaptability (B = 1.20, *p* < 0.001) on UWSS was positively significant. The model explained 66% of the variance in UWS. To test the effect of UWS on adult identity, an SEM analysis was performed with UWS as the independent variable and adult identity as the dependent variable. The result showed that the effect of UWSS on adult identity was also positively significant (B = 0.83, *p* < 0.001). The effect of UWSS remained significant (B = 0.39, *p* < 0.001) while controlling for career adaptability. This demonstrated UWSS’s incremental validity over general career success indicators of career adaptability in predicting an outcome unique to this particular stage, that is, the establishment of an adult identity.

**Table 7:** Means, standard deviations, and correlations.

Variable	*M*	*SD*	1	2	3	4	5	6	7	8
1. Gender	1.64	0.48								
2. Age	23.52	1.54	−0.03							
3. UWS	5.24	0.87	−0.14^**^	0.12^*^						
4. CIS	4.93	1.22	−0.12^*^	0.11^*^	0.91^**^					
5. IFI	5.21	0.97	−0.09	0.15^**^	0.80^**^	0.65^**^				
6. AW	5.37	0.79	−0.13^*^	0.06	0.87^**^	0.70^**^	0.62^**^			
7. CCF	5.45	1.1	−0.14^**^	0.10^*^	0.86^**^	0.75^**^	0.56^**^	0.68^**^		
8. Career adaptability	5.75	0.66	−0.08	0.09	0.66^**^	0.52^**^	0.51^**^	0.65^**^	0.60^**^	
9. Adult identity	3.62	0.63	−0.09	0.05	0.46^**^	0.42^**^	0.39^**^	0.43^**^	0.34^**^	0.41^**^

M and SD are used to represent mean and standard deviation, respectively. UWS = University-to-work Success; CIS = career insertion and satisfaction; IFI = income and financial independence; AW = adaptation to work; CCF = confidence in career future.

* Indicates *p* < 0.05.

** Indicates *p* < 0.01.

**Table 8:** Results of path analysis.

Predictor	UWS		Adult identity		Adult identity	
	*B*	*SE*	*B*	*SE*	*B*	*SE*
Gender	−0.13	0.08	−0.04	0.04	−0.07	0.08
Age	0.02	0.02	0.02	0.05	0.01	0.03
Career adaptability	1.20^***^	0.11			0.52^***^	0.14
UWS			0.83^***^	0.04	0.39^***^	0.10
*R* ^2^	0.66^***^		0.69^***^		0.75^***^	
*△R* ^2^					0.06^***^	

*N =* 384. SE: standard error. ***p < 0.001. UWS = University-to-work Success.

### 5.3 Discussion

The current study adapted the University to Work Success Scale (UWSS) for use in the Chinese context and examined the psychometric properties of the UWSS-Chinese version. Overall, the findings of the current study showed that the UWSS-Chinese version had good psychometric properties to be used in future studies and practice in China. The scale proved to have measurement invariance across genders, indicating that the scale can be used equivalently in measuring UWS for both genders transiting from university to work.

Several revisions are made to come up with the final Chinese version. To avoid having too few items after adaption for the Chinese context, the current study used all the original items generated in Oliveira et al. (2016)’s study instead of their final 22-item scale. Based on the contextual understanding of the university-to-work transition in China, three items that were inappropriate to reflect UWS in China were deleted from the Chinese scale. These items measured the fit between graduates’ field of degree and their work income and financial independence. Further, three items were deleted in the EFA step based on factor loadings and interpretation of the items. As a result, the UWSS-Chinese contains 24 items.

Concerning the factor structure, EFA results clearly showed four factors (career satisfaction, income and financial independence, confidence in career future, and adaptation to work) that are consistent with the original study. CFA results further confirmed the four factors. Also consistent with the original study, the second-order four-factor model and first-order four-factor model with CFA have a comparable level of model fit, suggesting that UWSS can be used to measure overall university-to-work success as well as the four aspects of success separately. Adding to the original scale development study, our study also tested the measurement invariance of UWSS across gender including configural, metric, and scalar invariance. Our results showed that the scale can be used equivalently in measuring UWS for both genders transitioning from university to work. This result added to the literature that discussed gender differences in work values and worker role identification in China (Skromme Granrose, 2007; Jiang and Yang, 2011; Yi et al., 2015). Our results showed that for the new generation of university graduates, female and male workers likely define school-to-work transition success in the same way, attaching equal importance to career satisfaction, independence, confidence in the future, and adaptation to work.

Our study also provided strong evidence that the UWSS-Chinese had good reliability and validity. It has an alpha coefficient of over 0.80 for all the subscales and the whole scale. Previously, the scale has shown convergent validity with one aspect of commonly used career success measurement, pay satisfaction. We provided additional construct evidence of the UWSS-Chinese by testing its relations with its theoretically proposed antecedents and outcomes. Past studies have shown that career adaptability is a critical psychosocial resource crucial for coping with the tasks of the university-to-work transition (De Oliveira et al., 2016; Harrison, et al., 2021; Zakkariya, et al., 2020). Our results confirmed that career adaptability significantly predicts UWSS scores. More importantly, based on theory and past empirical evidence, university-to-work transition as an important adult role transition should also contribute to adult identity development (Benson and Elder, 2011; Eliason et al., 2015; Piotrowski et al., 2020). Our results confirmed that the UWSS score predicts adult identity over and beyond the general career success indicator of career adaptability. This, in part, supports that UWSS captures transition outcomes specific to this life stage, adding evidence in support of its construct validity.

## 6 Limitations and future directions

This study had several limitations that warrant future examination in future studies. First, the nomological validity of the scale was tested with cross-sectional data. Thus, it cannot establish causality between UWS and the antecedents and outcome variable tested in the current study. Future studies should adopt a longitudinal design to examine more stringently the direction of the causality. Future studies should also expand the nomological network of the UWS. As discussed earlier, the development of the UWSS presents an important step forward toward a comprehensive and consensual measurement of university-to-work transition. With this measurement in place, future studies adopting different theoretical perspectives can adopt the same measurement to examine important propositions about the antecedents and outcomes of university to work success. For example, in terms of outcomes, future studies can examine if the UWSS score predicts long-term organizational adaptation and performance as the organizational socialization literature predicts (Bauer et al., 2007). In terms of antecedents, future studies can reexamine with this measurement if the school transition interventions promote successful school-to-work transition beyond obtaining employment, which was the current standard way of measuring UWS in the school-to-work-transition (STWT) literature (Koivisto et al., 2007). Second, this study did not assess the structural invariance of the relationships between UWS and other variables and the test–retest reliability of the UWSS–UWSS-Chinese version. Analyzing them is of value and could be carried out in a future study. Finally, despite acceptable theoretical and empirical evidence supporting the use of the scale in the current study, the reliability of the adult identity scale was not particularly high. Future studies may replicate the findings with the objective measurements for adulthood, such as the Makers of Adulthood scale, which has been applied in the Chinese context to cross-validate the current finding (Kuang et al., 2023).

## Data availability statement

The raw data supporting the conclusions of this article will be made available by the authors, without undue reservation.

## Ethics statement

The studies involving humans were approved by IRB of the School of Labor and Human Resources, Renmin University of China. The studies were conducted in accordance with the local legislation and institutional requirements. The participants provided their written informed consent to participate in this study.

## Author contributions

QJ: Conceptualization, Validation, Writing – original draft, Writing – review & editing. KY: Formal analysis, Funding acquisition, Methodology, Writing – original draft, Writing – review & editing.
